# Supplementary material for: Inflammatory factors and risk of lung adenocarcinoma: a Mendelian randomization study mediated by blood metabolites
Source: Front Endocrinol (Lausanne). 2024 Aug 27;15:1446863. doi: 10.3389/fendo.2024.1446863 (PMC11384989; doi:10.3389/fendo.2024.1446863)
Supplement: Supplementary file 1 [file DataSheet1.zip › supplementary files/S2/GCST90200241/sensitivity-analysis.pdf]

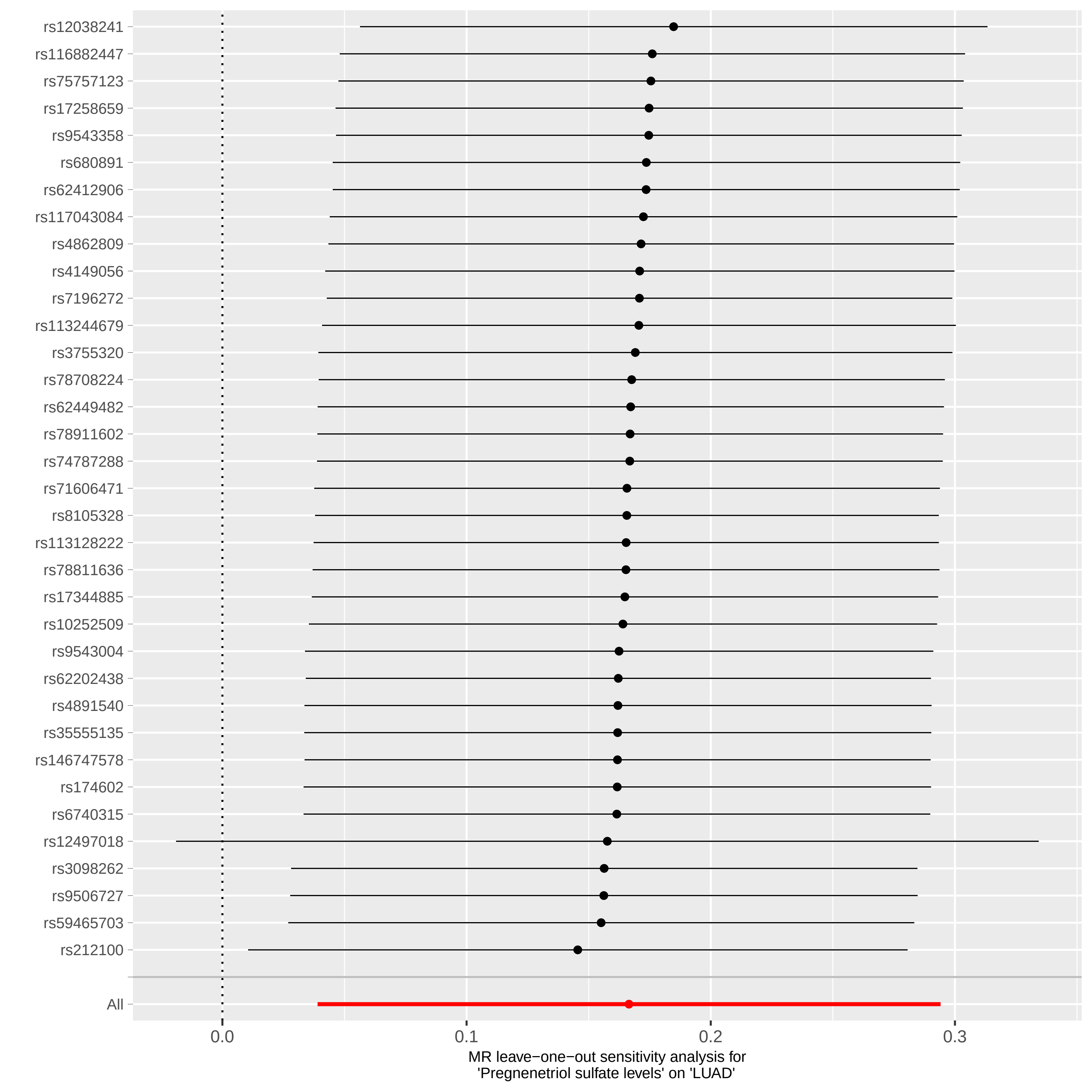
rs12038241
rs116882447
rs75757123
rs17258659
rs9543358
rs680891
rs62412906
rs117043084
rs4862809
rs4149056
rs7196272
rs113244679
rs3755320
rs78708224
rs62449482
rs78911602
rs74787288
rs71606471
rs8105328
rs113128222
rs78811636
rs17344885
rs10252509
rs9543004
rs62202438
rs4891540
rs35555135
rs146747578
rs174602
rs6740315
rs12497018
rs3098262
rs9506727
rs59465703
rs212100
All
0.0
0.1
0.2
0.3
MR leave−one−out sensitivity analysis for
'Pregnenetriol sulfate levels' on 'LUAD'
